# Systems Mapping of the New Zealand Free and Healthy School Lunch Programme: Perspectives from Lunch Providers

**DOI:** 10.3390/nu14204336

**Published:** 2022-10-17

**Authors:** Brittany Chote, David Rees, Boyd Swinburn, Pippa McKelvie-Sebileau, Rachael Glassey, David Tipene-Leach

**Affiliations:** 1 Research and Innovation Centre, Eastern Institute of Technology, Napier 4112, New Zealand; 2 Synergia Consulting Ltd., Auckland 1011, New Zealand; 3 School of Population Health, University of Auckland, Auckland 1023, New Zealand

**Keywords:** child nutrition, school meals, system dynamics, food security, policy

## Abstract

As part of the COVID-19 economic recovery package, the Aotearoa New Zealand Government rolled out a universal free and healthy lunch programme to the 25% least advantaged schools nationwide. This study explored experiences of school lunch providers in the Hawke’s Bay region. The aim was to create a systems map identifying points of intervention through which the lunch programme could be improved to meet the goal of reducing child food insecurity. Twelve lunch providers were interviewed to generate casual loop diagrams which were examined and integrated to form a single systems map. Seven themes arose during analysis: teacher support, principal support, nutrition guidelines and government support, supply chain, ingredient suppliers, student feedback and food waste. Teacher support was important for getting students to try new foods and eat the nutritious lunches. Principal support was a strong theme impacting opportunities for broader student engagement. This study employed systems science to highlight the importance of support from different stakeholders within the lunch programme to achieve the goal of reduced child food insecurity. Further work is needed to ensure the programme meets the wider goals of the government and community, and to determine the potential broader benefits of the programme.

## 1. Introduction

Free school meal programmes have been around for many decades across the globe, Sweden and Finland being the most cited examples in economically developed countries [1]. These Scandinavian countries offer free school meals on a universal basis, that is, every student is eligible to receive a free lunch [2]. Estonia, South Korea, the US, England and Scotland have all introduced variations of this free and universal approach but the concept is not widespread [3,4,5,6,7]. In 2019, Aotearoa New Zealand’s government announced a two-year pilot programme to explore the delivery of a free and healthy school lunch programme in a ‘whole of school’ approach to all students in the 25% least advantaged schools within several regions around the country [8]. As part of the COVID-19 economic recovery package in 2020, the lunch programme was expanded to include approximately 214,000 students in the least advantaged quartile of all state schools nationwide. The main aim of the programme, called *Ka Ora, Ka Ako* (being healthy enables learning), is to promote food security in school students, with additional goals including improved student health, wellbeing, concentration, behaviour and school achievement, reduced financial hardship in the home and increased school attendance [8].

Since the 1990s, schools in Aotearoa have had irregular government funding for nutrition initiatives, with food industry and centre-right politicians arguing that dietary choices are an individual’s responsibility [9]. The 2003 Healthy Eating Healthy Action and 2006 Mission-On campaigns providing nutrition resources and guidelines to help schools provide healthy food and beverage options for students were discontinued by the incoming government [10]. In 2012, the Children’s Commissioner’s Expert Advisory Group on Solutions to Child Poverty recommended a food in schools programme for the most disadvantaged schools but the government instead put funding into food programmes run by food manufacturing companies and private charities [11]. Ka Ora, Ka Ako is then, the biggest single intervention in children’s nutrition in Aotearoa New Zealand and interim results suggest it is positively impacting student hunger and wellbeing [12].

Select schools in the Hawke’s Bay region were part of the pilot for Ka Ora, Ka Ako, and now that the programme is fully rolled out, due to the high level of deprivation, 40% of school students in the region participate in the programme [8,13]. Children in Hawke’s Bay have poor vegetable intake and high rates of obesity compared to the national average [14]. Poor health indicators such as these led to the establishment of the Nourishing Hawke’s Bay (NHB): *He wairua tō te kai* (there is more to food than nutrition) initiative, a collaboration between Eastern Institute of Technology and the University of Auckland, School of Population Health [15]. The aim was to identify issues impacting the health of *tamariki* (children) and *rangatahi* (young people), and to work with schools to improve health outcomes, particularly for Indigenous Māori children. The community developed six *pou* (principles) to guide NHB actions: improve children’s *hauora* (health in the widest sense); start with schools; incorporate *mātauranga Māori* (Māori knowledge); improve *whānau* (family) food security; work with the community; and build on existing initiatives [15].

Ka Ora, Ka Ako began in Hawke’s Bay during the early stages of NHB. In response, community stakeholders decided that the top intervention priority for NHB was to ensure that the programme met the recommendations of the six pou and improved health outcomes for Indigenous Māori and other disadvantaged children. The objectives for this intervention were (1) to co-create with school lunch providers, a systems map of Ka Ora, Ka Ako processes to identify the issues and opportunities for improvement, (2) to assess the impact of Ka Ora, Ka Ako on whānau food security and (3) to create a baseline measure for longitudinal assessment of the impact of Ka Ora, Ka Ako school lunches on children’s health.

The aim of this paper is to describe the systems map which was created by interviewing school lunch providers to identify points of intervention through which this new programme can be improved so that it meets the goal of promoting food security in school students.

## 2. Materials and Methods

### 2.1. Study Design

This study employed a systems science approach which aims to understand the mechanisms driving behaviours within a system by identifying the structure of causal relationships and feedback loops. The application of systems thinking in public health nutrition is increasing [16,17], including significant research in obesity prevention [18,19,20,21,22,23,24,25]. In the qualitative use of system dynamics (SD), causal loop diagrams (CLDs) are developed with community participants to understand how key elements in a system interact and feedback upon each other to produce certain behaviours. This approach has been found to be highly effective in engaging communities to explore barriers to action and developing ‘bottom-up’ solutions that are acceptable to the community and sustainable within an existing system [26]. CLDs assist community stakeholders to express their understanding of the system surrounding an issue, regardless of prior experience with systems thinking [26].

Data collection was in the form of semi-structured interviews which were analysed to generate CLDs consisting of three basic elements: variables, connections between those variables and feedback loops generated by those connections. These together draw a picture of the causal structure of a system [27]. School lunch providers were interviewed to understand how different school lunch programmes were functioning, and to determine what was working and what the challenges were. In addition, wider impacts of the programme were explored, as well as possible ways to improve the programme. Internal (on-site cooks) and external (off-site providers) lunch suppliers were interviewed between February and June 2022. Ka Ora, Ka Ako allows schools to either use on-site cooks (who are often members of the community with chef or catering experience) or external off-site providers (who are mostly private catering businesses), or a mixture of both, to provide the lunches [8]. Each supplier was interviewed twice (except for one who was interviewed once) either in person or in an online call. Each provider also completed a short questionnaire about their school lunch programme either before or during the first interview to provide background information on their lunch delivery system. The questionnaire asked when the provider joined the Ka Ora, Ka Ako programme, approximately how many lunches they made each day, where they purchased their produce, bread, meat and packaging from, and for internal providers, if their school grew any of their own ingredients. This study was approved by the Research Ethics and Approvals Committee of the Eastern Institute of Technology (reference number: NO11131221). Informed consent was obtained from all participants.

### 2.2. Recruitment and Data Collection

Eight external suppliers and eight internal suppliers from Hawke’s Bay were invited via email to take part in this research. These suppliers were selected based on previous engagement with NHB or through recommendations from local schools. Follow up emails and phone calls were made where there had been no response to the initial email invite.

The first interviews were semi-structured with the focus being an overview of the lunch providers’ operations from procuring ingredients through to preparation, transport, consumption of the lunches by the students, and the waste stream at the end. Each participant was also specifically asked what they would change about the Ka Ora, Ka Ako programme, if anything. The interviews lasted 30–70 min, and were audio-recorded. Second interviews were held with each participant, these lasted a similar length of time and were also audio-recorded. The purpose of the second interviews were for participants to provide feedback on the CLDs generated from the first interviews and confirm if the researcher had interpreted the information correctly. They were also an opportunity for a richer discussion of key ideas and to fill information gaps.

### 2.3. Data Analysis

The interview recordings from the first interviews were reviewed to identify key causal relationships discussed by each participant. A cognitive map which described each of these relationships was created for each lunch provider in KUMU (kumu.io). The individual maps were then consolidated into a combined map where themes were identified and constructs were coded against each of these themes. Themes were then pulled out to create a thematic map, from which CLDs were identified. Each CLD was examined in relation to the other CLDs and were integrated into one diagram. Not all causal relationships identified in the interviews were feedback loops but were still represented in the diagram where appropriate. The CLDs were centered around the ‘students eating nutritious meals’ component because the main aim of Ka Ora, Ka Ako is to promote food security in school children. Access to nutritious food is an essential characteristic in the definition of ‘food security’ [28]. System dynamics convention was used to explain the causal relationships [29]. CLDs were labelled as ‘reinforcing’ (R), indicating loops where change is compounded, often called ‘virtuous’ or ‘vicious’ cycles; or ‘balancing’ (B), where patterns are held in balance as one connection reduces the effect of another, much like a thermostat. Positive polarity (blue lines) indicate a positive relationship between the two variables (i.e., as one increases the other increases or as one decreases the other decreases), and a negative polarity (red lines) indicate an inverse relationship between the two variables (i.e., as one increases the other decreases or vice versa). The CLDs were presented to the wider research team for discussion and to determine the focus for the second interviews. The diagrams and naming of each construct were carefully reviewed and modified following this discussion. These modified CLDs were then presented to the participants in the second interviews. Modifications and refinements were made to the CLDs based on feedback from the second interviews.

## 3. Results

Twelve lunch suppliers were interviewed, six internal and six external. One of the internal suppliers was also the principal of the school. Five internal cooks provided lunch for primary/intermediate schools (years 1–8), and one provided lunch for a high school (years 9–12) and a primary school. One external supplier provided lunch to primary/intermediate schools, two provided to intermediate only schools (years 7–8) and two provided to both primary and high schools. The number of lunches made each day by the internal cooks ranged from about 100 to 1000, whereas the number of lunches the external providers made ranged from approximately 300 to 2800.

**Figure 1:**
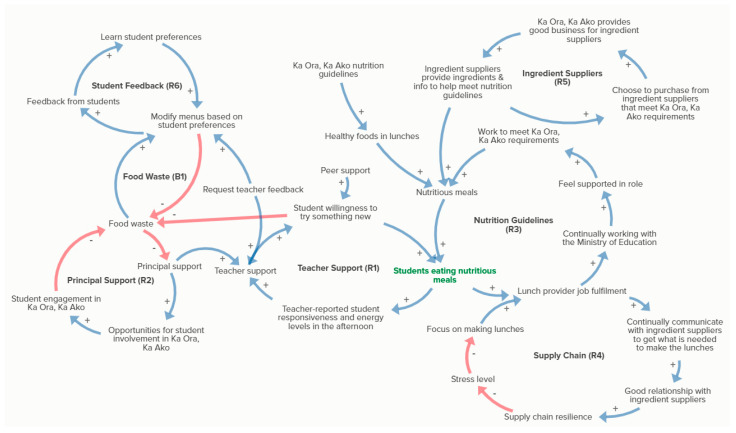
Causal loop diagram from interviews with Ka Ora, Ka Ako lunch suppliers. Positive polarity (blue lines) indicate a positive relationship between the two variables (as one increases the other increases or as one decreases the other decreases); a negative polarity (red lines) indicate an inverse relationship between the two variables (as one increases the other decreases or vice versa).

The final causal loop map comprised of six reinforcing loops and one balancing loop (Figure 1). These related to: teacher support, principal support, the Ka Ora, Ka Ako nutrition guidelines and government support, supply chain, ingredient suppliers, student feedback and food waste.

### 3.1. Teacher Support

One of the strongest themes to emerge from the interviews was teacher support. This was expressed in various ways, for example, teachers eating the lunches with students, teachers encouraging students to try the lunches or serving the lunches to students. “Teacher support” led to “student willingness to try something new” which was important, as many of the foods were foreign to students at the beginning of the programme.

*“[The teachers] get what the kids get to eat because they’re the role models. They never used to, we just used to do the kids. Then we were like the kids aren’t gonna eat it cause we need them to be in the waka (canoe) with them.”—Internal supplier*

“Peer support” was also an influencer of “student willingness to try something new”, particularly for lunch suppliers of older intermediate and high school students.

Because of “student willingness to try something new” there were more “students eating nutritious meals”, i.e., the lunches. This led to increased “teacher-reported student responsiveness and energy levels in the afternoon” which helped the teachers with their job as educators and led to increased “teacher support” for Ka Ora, Ka Ako. In addition, because there was greater “student willingness to try something new” there was less “food waste”.

*“If you look at the scrap bins at the end of lunch you can see [which teacher] is engaged and who is not…it is clearly obvious.”—External supplier*

Variables that also led to an increase in “teacher support” were “principal support” of Ka Ora, Ka Ako and “requesting teacher feedback” on the lunches.

### 3.2. Principal Support

Principal support was a strong theme throughout the interviews. Principals were considered the main agents of change within a school and determined the school culture. Where “principal support” for Ka Ora, Ka Ako was clear, either through leading the programme in their school, or giving teachers and lunch providers the time and resources needed to incorporate the lunches into school life, there were improved “opportunities for student involvement in Ka Ora, Ka Ako”. For example, students helping with menu design and lunch preparation, and incorporating nutrition into the curriculum. This involvement increased “student engagement in Ka Ora, Ka Ako” and reduced “food waste” as the students were more interested in the lunches.

*“Where we were having things like meatball subs, the bread was still within the guidelines but the kids didn’t like the quality of it so we do meatballs on a base salad now. So they created that salad and they’ve had to do the surveys around you know the lettuce or spinach or whatever but in the same note they’re all eating it.”—Internal supplier*

### 3.3. Nutrition Guidelines and Government Support

The nutrition guidelines loop is about having a good working relationship with the Ministry of Education (MoE). “Continually working with the Ministry of Education” led to lunch providers “feeling supported in their role” and “working to meet the Ka Ora, Ka Ako requirements”. This enabled them to produce “nutritious meals” and therefore get “students to eat nutritious meals”, leading to “lunch provider job fulfilment” which in turn encouraged them to “continually work with the Ministry of Education”.

Participants discussed how the “Ka Ora, Ka Ako nutrition guidelines” were a key element of the programme and led to the inclusion of “healthy foods in lunches” and therefore the creation of “nutritious meals”:

*“We would just be winging it…I mean we know what’s healthy and that but we don’t know exactly how much protein a child needs at this age or exactly how much fibre.”—Internal supplier*

There were a few external suppliers that found the guidelines time consuming and expensive but the majority of participants agreed it was good to have targets to work towards, even if they were still on the journey to reach them.

*“My attitude towards it is yes I will try my best to follow the guidelines but it’s a gradual process, and the Ministry lady agrees with me.”—External supplier*

### 3.4. Supply Chain

Creating “good relationships with ingredient suppliers” was considered an important element in improving “supply chain resilience”. These relationships were created and maintained through ongoing communication.

*“Recently the owner [of the produce company] has been doing the deliveries, so has been out delivering himself which is good relationship building, like way back in 2019 before it even started I went down and met him…this builds that connection. But even his driver, I’m quite well connected with him…if I’ve missed something out or decided last minute I want some of this I feel I can always just ring”—Internal supplier*

“Supply chain resilience” in turn reduced “stress levels” and allowed for suppliers to “focus on making lunches”. This helped with “lunch provider job fulfilment” and “continued communication with ingredient suppliers to get what is needed to make the lunches”.

### 3.5. Ingredient Suppliers

It was raised by several participants that “Ka Ora, Ka Ako provided good business for ingredient suppliers” which has in turn prompted “ingredient suppliers to provide ingredients and information to help meet the nutrition guidelines”. For example, ingredient suppliers putting nutrition information on their websites that lunch suppliers need to plan menus and having functional apps that can be used to place orders. Because these ingredient suppliers were meeting the needs of lunch suppliers, lunch suppliers continued to “choose to purchase from ingredient suppliers that meet the Ka Ora, Ka Ako requirements”.

*“…when the guidelines changed we needed more fibre in [our bread]. We went to the bread company and said ‘we can’t use your bread anymore, we’re out’. They said ‘why’ and we told them, and they were like ‘don’t worry about it, we’ll fix it’, so they put more fibre in their bread—External supplier*

*“We’ve spent quite a comprehensive amount of time talking to suppliers and getting them to make goods that fit the criteria, so sodium content, fat content, everything.”—External supplier*

Additionally, because “ingredient suppliers provide ingredients and information to help meet the nutrition guidelines”, “nutritious lunches” could be created which was an essential construct for achieving the goal of “students eating nutritious meals”

### 3.6. Student Feedback

External suppliers were able to get student feedback about the lunches through written notes from students, surveys or when they were on school grounds to drop off the lunches. Internal providers were able to build relationships with students through their presence at school. These examples of getting “feedback from students” meant suppliers could “learn student preferences” and therefore “modify menus based on student preferences”.

*“We always get notes like ‘Hi [supplier’s name], lunch today was yum can we have it again?’. Otherwise we’ll have ones like ‘never give us this again with beetroot in it, it was gross’…that’s what we want from them, I was like if something’s yuck we don’t know what we don’t know so you need to tell us.”—External supplier*

“Requesting teacher feedback” also fed into this causal loop, assisting with “menu modification based on student preferences”.

### 3.7. Food Waste

“Modifying menus based on student preferences” led to a reduction in “food waste”. As “food waste” was reduced so did the need to “modify menus based on student preferences” because the students were enjoying the lunches more. As previously discussed “student engagement in Ka Ora, Ka Ako” and “student willingness to try something new” also led to reduced “food waste”.

## 4. Discussion

Interviews with internal and external Ka Ora, Ka Ako lunch providers from around Hawke’s Bay enabled the creation of a systems map of the programme which highlighted seven key themes that are essential to the ‘reduction of child food insecurity by providing access to a nutritious lunch every day’. The themes were teacher support, principal support, nutrition guidelines and government support, supply chain, ingredient suppliers, student feedback and food waste. The causal loop diagram illustrated the key variables that affect the success of Ka Ora, Ka Ako. For example, the importance of good working relationships between the MoE and lunch suppliers to ensure that the nutrition guidelines are met, and how teacher support can influence students’ willingness to try new foods and therefore consume the nutritious lunches on offer. Altering one of these constructs will have potential consequences throughout the system and on the outcomes of Ka Ora, Ka Ako.

Systems science methodology is a novel approach in school meal programme research, although, several other studies have interviewed school cooks and caterers in Europe and North America using other qualitative methods of analysis. Chambers and colleagues used Normalisation Process Theory to investigate the implementation of universal, free meals in Scottish primary schools [5]. Similar to our work, they found that teacher and principal support were vital components to the uptake of school meals by students. Where there was perceived tension between lunch staff and school leaders, implementation of the school lunch programme was hampered. In contrast, it was perceived that if teachers were present in the dining hall, students would be much more likely to try the lunch. Data from school administrators in Norway also suggests that teacher presence during lunch had the additional benefit of strengthening teacher-student relationships [30]. Research from the US investigating the barriers and facilitators of the implementation of the new National School Lunch programme guidelines from the perspective of food service directors highlighted that teachers need to participate in the food programme [31]. Districts that reported greater teacher support reported greater success in the implementation of healthier nutrition guidelines. A key component of building teacher support was developing relationships between teachers and suppliers. This is consistent with what we found, whereby engaging the teachers and getting their perspective on the programme was considered a way of increasing teacher support.

Having strong nutrition guidelines as the backbone of school lunch programmes has become increasingly common [4,32,33,34]; however, student acceptance of these healthier foods can be a challenge. Ka Ora, Ka Ako has a strict nutrition policy which is considered by many lunch providers to be a helpful tool to improve meals over time. However, some external providers found the guidelines difficult to meet within their budget, menus were time consuming to put together and student acceptance of the healthier food was low. International research also illustrates conflicting opinions amongst school meal providers, with some reporting increased costs and lower student acceptance associated with healthier guidelines, whereas others say, with some creativity, healthy menus can be created that are still familiar enough that students are willing to try the food, therefore reducing food waste [7,31,35]. Our research shows that building relationships with students and obtaining their feedback helps improve student consumption of nutritionally compliant lunches as students are very honest about their food preferences and can provide useful feedback on menus, particularly as they learn more about which foods are healthy and which are not.

Food preferences, eating autonomy, social pressures, the presence of food outlets near school grounds, cultural appropriateness, providing more time to eat lunch and regularity of the programme all influence the uptake of school meals [35,36,37,38,39,40]. In addition to these individual factors influencing the likelihood of student participation, the interaction of such constructs is important. In their Norwegian study, Mauer and colleagues [35] suggested that for students the popularity of the food, combined with “social eating” and the establishment of new routines around school meals, collectively counterbalanced the appeal of buying food offsite. It was the interaction of these individual constructs that was just as important as the constructs themselves. This reinforces our findings using systems science, highlighting that a variable at first glance can appear to exist independently, when in fact it is part of a system where multiple variables interact with each other.

This study has allowed us to understand the system dynamics of the Ka Ora, Ka Ako programme and what is required to meet the government goal of reducing food insecurity in school children through the provision of a nutritious lunch each day. These findings can be applied at both a school and government policy level. For example, within schools, staff can redesign food policies to support the Ka Ora, Ka Ako programme. We know that currently school food policies in Aotearoa New Zealand are not being used effectively to improve the food environment but have the potential to do so [41,42]. At a government level, this research can guide the improvement of Ka Ora, Ka Ako through increased information sharing between principals and the MoE, as we have identified that principals are the major agents of change within a school and influence the level of teacher support for the programme. Government could also assist in the development of partnerships between schools and food and nutrition education programmes that align with the Ka Ora, Ka Ako nutrition guidelines. Our participants highlighted that engaging students in the lunch programme through cooking, classroom education and school gardens may improve up-take of the lunches. Food and nutrition education programmes are well placed to assist with this [43]. The next challenge is to identify ways that Ka Ora, Ka Ako can meet the community’s pou, identified in previous NHB research [15]. The pillars of working with schools, improving food security and in-part, improving children’s hauora are being met by the programme. Incorporating mātauranga Māori is not currently a priority for Ka Ora, Ka Ako and NHB is investigating this further, as is the MoE. Working with community members to develop a cohesive approach to connect people and projects should also be a focus of future inquiry to get the most out of the lunch programme. Consultation with all stakeholders, including education staff, lunch providers and whānau, needs to be a continuous process. Chambers et al., highlight that policy makers responsible for new school lunch programmes need to invest in the relational work between stakeholders to ensure the right practices are in place for long term success [5]. Making change within an educational context also comes with specific challenges, most notably “policy and strategy overload” which results in staff not having sufficient time to fully engage in change [44]. Further work within the community is required to identify ways of linking their goals with this government programme that is also bound by its placement within the education sector.

### 4.1. Future Research

In addition to the casual loop diagrams presented in Figure 1, relationships and ideas were discussed with participants during the interviews that were not included in the map as there was not sufficient data to build feedback loops. These ideas either fed into or were potential spinoffs of the causal loops. Examples include the potential to incorporate elements of mātauranga Māori and *tikanga Māori* (Māori ways of doing things such as saying grace before a meal, eating together) into Ka Ora, Ka Ako, further educational impacts of the programme if it connected with the nutrition education curriculum, and the economic elements that are associated with the programme, such as the economic viability of Ka Ora, Ka Ako and the creation of jobs across the system. NHB is presently developing a quantitative SD model to delve deeper into these ideas and to measure the potential impact of their inclusion in the Ka Ora, Ka Ako system, using the data collected in this initial work as a base for that model. The project is also undertaking a policy pathways analysis to identify specific policy actions that could widen the impact of Ka Ora, Ka Ako across multiple sectors, is investigating how it can contribute to regional food system transformation and exploring how Ka Ora, Ka Ako links to different parts of the curriculum, for example, its association with literacy. Further research into these ideas will help to determine the full potential of the lunch programme and whether or not it can benefit children beyond the initial goals set by the government.

### 4.2. Strengths and Limitations

Only three of the suppliers in the study provided lunches to high schools. From these interviews there were indications that they had different experiences to those supplying primary and intermediate schools, however a larger number of participants would be required to investigate this further. Due to the relatively small number of participants and geographic area covered caution is needed in generalising these findings beyond Hawke’s Bay. However, this work provides novel insights into how the school lunch programme is functioning as a whole for lunch providers; considering how constructs interact with one other, not just how they stand in isolation through the use of systems science. This is the first piece of research working with lunch providers from the New Zealand school lunch programme. Because lunch providers are the stakeholders on the ground their perspective is vital for the success of Ka Ora, Ka Ako.

## 5. Conclusions

Aotearoa New Zealand does not have a robust history of healthy food and food security interventions in schools. The Ka Ora, Ka Ako school lunch programme, rolled out to low advantage schools nationwide during the COVID-19 pandemic, is the first systematic attempt to promote food security in school children. Lunch providers are the workers on the ground keeping this programme running, therefore understanding their perspective of how Ka Ora, Ka Ako is functioning is essential. We have used systems science to create a visual representation of the key themes to success from the outlook of these providers. It is important to consider how the key themes identified work as feedback loops within themselves but also how they interact with each other when determining the long-term structure of the lunch programme. Further research will be able to establish how other factors can support the causal relationships identified here, therefore ensuring school children in Aotearoa New Zealand experience improved food security and as well potentially broader benefits in the future.

## Data Availability

Not applicable.

## References

[1] Waling M., Olafsdottir A.S., Lagström H., Wergedahl H., Jonsson B., Olsson C., Fossgard E., Holthe A., Talvia S., Gunnarsdottir I. (2016). School meal provision, health, and cognitive function in a Nordic setting—The ProMeal-study: Description of methodology and the Nordic context. Food Nutr. Res..

[2] Ray C., Roos E., Brug J., Behrendt I., Ehrenblad B., Yngve A., Velde S.J.T. (2013). Role of free school lunch in the associations between family-environmental factors and children’s fruit and vegetable intake in four European countries. Public Health Nutr..

[3] Cohen J.F.W., Hecht A.A., McLoughlin G.M., Turner L., Schwartz M.B. (2021). Universal school meals and associations with student participation, attendance, academic performance, diet quality, food security, and body mass index: A systematic review. Nutrients.

[4] Gaddis J.E., Jeon J. (2020). Sustainability transitions in agri-food systems: Insights from South Korea’s universal free, eco-friendly school lunch program. Agric. Hum. Values.

[5] Chambers S., Boydell N., Ford A., Eadie D. (2020). Learning from the implementation of Universal Free School Meals in Scotland using Normalisation Process Theory: Lessons for policymakers to engage multiple stakeholders. Food Policy.

[6] Forrestal S., Potamites E., Guthrie J., Paxton N. (2021). Associations among food security, school meal participation, and students’ diet quality in the first School Nutrition and Meal Cost Study. Nutrients.

[7] Zuercher M.D., Cohen J.F.W., Hecht C.E., Hecht K., Ritchie L.D., Gosliner W. (2022). Providing school meals to all students free of charge during the COVID-19 pandemic and beyond: Challenges and benefits reported by school foodservice professionals in California. Nutrients.

[8] Ministry of Education (2022). Ka Ora, Ka Ako, Healthy School Lunches Programme.

[9] Swinburn B., Wood A. (2013). Progress on obesity prevention over 20 years in Australia and New Zealand. Obes. Rev..

[10] Pledger M., Black J., Cumming J., McDonald J. (2010). 2009 School and Early Childhood Education Services Food and Nutrition Environment Survey: Phase III Report. Health Services Research Centre.

[11] Wynd D., O’Brien M. (2014). Food in schools: Targeting versus the right to food. N. Z. Med. J..

[12] Vermillion Peirce P., Blackie E., Morris M., Jarvis-Child B., Engelbertz S. (2021). New Zealand Healthy School Lunch Pilot Ka Ora, Ka Ako Interim Evaluation.

[13] Ministry of Education School Rolls—Interactive Dashboard. https://www.educationcounts.govt.nz/statistics/school-rolls.

[14] Ministry of Health Regional Data Explorer 2017–20: New Zealand Health Survey. https://minhealthnz.shinyapps.io/nz-health-survey-2017-20-regional-update/_w_34fe3939/#!/.

[15] McKelvie-Sebileau P., Rees D., Swinburn B., Gerritsen S., D’Souza E., Tipene-Leach D. (2021). Combining Cognitive Mapping and Indigenous knowledge to improve food environments in regional New Zealand. Health Promot. J. Aust..

[16] Gerritsen S., Renker-Darby A., Harré S., Rees D., Raroa D.A., Eickstaedt M., Sushil Z., Allan K., Bartos A.E., Waterlander W.E. (2019). Improving low fruit and vegetable intake in children: Findings from a system dynamics, community group model building study. PLoS ONE.

[17] Mui Y., Ballard E., Lopatin E., Thornton R.L.J., Porter K.M.P., Gittelsohn J. (2019). A community-based system dynamics approach suggests solutions for improving healthy food access in a low-income urban environment. PLoS ONE.

[18] Maitland N., Wardle K., Whelan J., Jalaludin B., Creighton D., Johnstone M., Hayward J., Allender S. (2021). Tracking implementation within a community-led whole of system approach to address childhood overweight and obesity in south west Sydney, Australia. BMC Public Health.

[19] Owen B., Brown A.D., Kuhlberg J., Millar L., Nichols M., Economos C., Allender S. (2018). Understanding a successful obesity prevention initiative in children under 5 from a systems perspective. PLoS ONE.

[20] Brennan L.K., Sabounchi N.S., Kemner A.L., Hovmand P. (2015). Systems thinking in 49 communities related to healthy eating, active living, and childhood obesity. J. Public Health Manag. Pract..

[21] Jenkins E., Lowe J., Allender S., Bolton K.A. (2020). Process evaluation of a whole-of-community systems approach to address childhood obesity in western Victoria, Australia. BMC Public Health.

[22] Malakellis M., Hoare E., Sanigorski A., Crooks N., Allender S., Nichols M., Swinburn B., Chikwendu C., Kelly P.M., Petersen S. (2017). School-based systems change for obesity prevention in adolescents: Outcomes of the Australian Capital Territory ‘It’s Your Move!’. Aust. N. Z. J. Public Health.

[23] Waterlander W.E., Singh A., Altenburg T., Dijkstra C., Pinzon A.L., Anselma M., Busch V., van Houtum L., Emke H., Overman M.L. (2021). Understanding obesity-related behaviors in youth from a systems dynamics perspective: The use of causal loop diagrams. Obes. Rev..

[24] Bagnall A.-M., Radley D., Jones R., Gately P., Nobles J., Van Dijk M., Blackshaw J., Montel S., Sahota P. (2019). Whole systems approaches to obesity and other complex public health challenges: A systematic review. BMC Public Health.

[25] Brown A.D., Whelan J., Bolton K.A., Nagorcka-Smith P., Hayward J., Fraser P., Strugnell C., Felmingham T., Nichols M., Bell C. (2022). A theory of change for community-based systems interventions to prevent obesity. Am. J. Prev. Med..

[26] Hovmand P.S. (2014). Community Based System Dynamics.

[27] Barbrook-Johnson P., Penn A.S. (2022). Systems Mapping: How to Build and Use Causal Models of Systems.

[28] FAO (2001). The State of Food Insecurity in the World 2001.

[29] McKelvie-Sebileau P., Rees D., Tipene-Leach D., D’Souza E., Swinburn B., Gerritsen S. (2022). Community co-design of regional actions for children’s nutritional health combining Indigenous knowledge and systems thinking. Int. J. Environ. Res. Public Health.

[30] Heim G., Thuestad R.O., Molin M., Brevik A. (2022). Free school meal improves educational health and the learning environment in a small municipality in Norway. Nutrients.

[31] Tabak R.G., Moreland-Russell S. (2015). Food service perspectives on National School Lunch Program implementation. Health Behav. Policy Rev..

[32] Au L.E., Gurzo K., Gosliner W., Webb K.L., Crawford P.B., Ritchie L.D. (2018). Eating school meals daily is associated with healthier dietary intakes: The Healthy Communities Study. J. Acad. Nutr. Diet..

[33] Holthe A., Larsen T., Samdal O. (2011). Understanding barriers to implementing the Norwegian national guidelines for healthy school meals: A case study involving three secondary schools. Matern. Child Nutr..

[34] Evans C.E.L., Melia K.E., Rippin H.L., Hancock N., Cade J. (2020). A repeated cross-sectional survey assessing changes in diet and nutrient quality of English primary school children’s packed lunches between 2006 and 2016. BMJ Open.

[35] Mauer S., Torheim L.E., Terragni L. (2022). Children’s participation in free school meals: A qualitative study among pupils, parents, and teachers. Nutrients.

[36] Sahota P., Woodward J., Molinari R., Pike J. (2014). Factors influencing take-up of free school meals in primary- and secondary-school children in England. Public Health Nutr..

[37] Bailey-Davis L., Virus A., McCoy T.A., Wojtanowski A., Vander Veur S.S., Foster G.D. (2013). Middle school student and parent perceptions of government-sponsored free school breakfast and consumption: A qualitative inquiry in an urban setting. J. Acad. Nutr. Diet..

[38] Waddingham S., Shaw K., Van Dam P., Bettiol S. (2018). What motivates their food choice? Children are key informants. Appetite.

[39] Sobek C., Ober P., Abel S., Spielau U., Kiess W., Meigen C., Poulain T., Igel U., Vogel M., Lipek T. (2021). Purchasing behavior, setting, pricing, family: Determinants of school lunch participation. Nutrients.

[40] Cohen J.F.W., Hecht A.A., Hager E.R., Turner L., Burkholder K., Schwartz M.B. (2021). Strategies to improve school meal consumption: A systematic review. Nutrients.

[41] Chote B., McKelvie-Sebileau P., Swinburn B., Tipene-Leach D., D’Souza E. (2022). Culture of healthy eating and food environments, policies, and practices in regional New Zealand schools. Int. J. Environ. Res. Public Health.

[42] D’Souza E., Vandevijvere S., Swinburn B. (2022). The healthiness of New Zealand school food environments: A national survey. Aust. N. Z. J. Public Health.

[43] Koch P., McCarthy J., Raffel C., Gray H.L., Guerra L.A. (2020). Expanding and enhancing food and nutrition education in New York City public schools: An examination of program characteristics and distribution. Nutrients.

[44] Wood P. (2017). Overcoming the problem of embedding change in educational organizations: A perspective from Normalization Process Theory. Manag. Educ..

